# Evaluating Receptor-Specific Fresh Specimen Staining for Tumor Margin Detection in Clinical Breast Specimens

**DOI:** 10.1007/s11307-022-01771-9

**Published:** 2023-06-23

**Authors:** Brook K. Byrd, Wendy A. Wells, Rendall R. Strawbridge, Connor W. Barth, Kimberley S. Samkoe, Summer L. Gibbs, Scott C. Davis

**Affiliations:** 1 Thayer School of Engineering, Dartmouth College, Hanover, NH 03755 USA; 2 Department of Pathology and Laboratory Medicine, Dartmouth Hitchcock Medical Center, Lebanon, NH 03766 USA; 3 Biomedical Engineering Department, Oregon Health & Science University, Portland, OR 97201 USA

**Keywords:** Molecular imaging, Fluorescence-guided surgery (FGS), Breast cancer surgery, Tumor margins, HER2, Fluorescence imaging

## Abstract

**Purpose:**

Reliable and rapid identification of tumor in the margins of breast specimens during breast-conserving surgery to reduce repeat surgery rates is an active area of investigation. Dual-stain difference imaging (DDSI) is one of many approaches under evaluation for this application. This technique aims to topically apply fluorescent stain pairs (one targeted to a receptor-of-interest and the other a spectrally distinct isotype), image both stains, and compute a normalized difference image between the two channels. Prior evaluation and optimization in a variety of preclinical models produced encouraging diagnostic performance. Herein, we report on a pilot clinical study which evaluated HER2-targeted DDSI on 11 human breast specimens.

**Procedures:**

Gross sections from 11 freshly excised mastectomy specimens were processed using a HER2-receptor-targeted DDSI protocol shortly after resection. After staining with the dual-probe protocol, specimens were imaged on a fluorescence scanner, followed by tissue fixation for hematoxylin and eosin and anti-HER2 immunohistochemical staining. Receiver operator characteristic curves and area under the curve (AUC) analysis were used to assess diagnostic performance of the resulting images. Performance values were also compared to expression level determined from IHC staining.

**Results:**

Eight of the 11 specimens presented with distinguishable invasive ductal carcinoma and/or were not affected by an imaging artifact. In these specimens, the DDSI technique provided an AUC = 0.90 ± 0.07 for tumor-to-adipose tissue and 0.81 ± 0.15 for tumor-to-glandular tissue, which was significantly higher than AUC values recovered from images of the targeted probe alone. DDSI values and diagnostic performance did not correlate with HER2 expression level, and tumors with low HER2 expression often produced high AUC, suggesting that even the low expression levels were enough to help distinguish tumor.

**Conclusions:**

The results from this preliminary study of rapid receptor-specific staining in human specimens were consistent with prior preclinical results and demonstrated promising diagnostic potential.

## Introduction

Breast conserving surgery (BCS) is a common procedure used to treat over 60% of patients diagnosed with breast cancer in the USA [1]. This procedure is designed to completely remove malignant lesions while preserving as much normal breast tissue as possible. Yet, ensuring a complete resection is a persistent clinical challenge, and studies have shown that tumor remains on the cut surface of the resected specimen in 15 to 30% of BCS procedures [2–5]. The determination of a positive margin is typically made via pathological analysis of the excised specimen several days after the surgical procedure, and often prompts a second surgical procedure to try to remove residual tumor tissue. These re-excision surgeries can take the form of a breast-conserving margin-widening procedure or radical mastectomy, depending on the diagnosis after the primary surgery and criteria applied by the surgeon [6–8]. A margin widening procedure can preserve much of the remaining breast tissue, but localizing residual disease can sometimes be challenging due to tissue displacement and inflammation. Even when successful, these procedures cause patient stress, increase morbidity, and burden the healthcare system, and thus, reducing the rates of incomplete resection during the primary surgery is a widely recognized goal.

In this context, intra-surgical margin assessment strategies have been translated to clinical practice or are in various stages of clinical development. Technologies currently deployed for intraoperative margin assessment include specimen mammography, which is widely considered standard of care [9, 10], frozen sectional analysis [11–13], and touch prep cytology [11–13]. These technologies have not fully addressed the re-excision problem, prompting broad efforts to develop new approaches to reducing re-excision rates. Many of the technologies under development leverage optical imaging strategies, including imaging tumoral changes in tissue scatter properties using structured light [14–17] or optical coherence tomography [18–21], leveraging changes in Raman signatures using either endogenous [22–25] or exogenous markers[26–28], or imaging fluorescent contrast agents administered in vivo [29–34], or on tissue specimens [35–37]. In vivo fluorescence imaging is a compelling approach which aims to reveal residual tumor sites directly in the surgical cavity, and one of the more advanced efforts recently reported promising data from a multicenter clinical trial [34].

Topical staining and imaging of fresh tissue specimens with tumor-specific probes is another active area of inquiry. This strategy aims to rapidly stain and image the surface of the specimen to identify positive margins during surgery, facilitating further excision to remove residual tumor. The approach precludes the need to establish the in vivo safety profiles of novel contrast agents, and enables the multiplexing of probes targeting various biomarkers. Multiple groups have shown that including a spectrally distinct non-targeted isotype probe in the staining solution, acquiring images of both probes in separate channels, and mathematically comparing the two channels helps compensate for imaging system inhomogeneity and produce images of receptor-specific tumor contrast [27, 28, 35–39]. This strategy, termed dual-probe difference specimen imaging (DDSI) in our lab, has been applied using antibodies labeled with surface-enhanced Raman scattering (SERS) nanoparticles [26–28], quantum dots [40] or with spectrally distinct fluorophores [35–37] to target various biomarkers, including ERBB1 (EGFR), ERBB2 (HER2), estrogen receptor, and CD44. Through a series of preclinical studies, we showed that fluorophore-based DDSI of EGFR or HER2 provided high tumor-to-normal diagnostic performance, with an area under the curve (AUC) from receiver operator characteristic (ROC) curves routinely > 0.95 [35–38]. We also used preclinical models to optimize the staining protocol, reducing the tissue processing time to 6 min [37]. To date, however, fluorophore-based DDSI had not been evaluated in human breast specimens.

Building upon our prior preclinical work, we conducted an observational study to evaluate HER2-targeted DDSI imaging in human breast specimens. Mastectomy specimens from 10 patients were gross-sectioned, stained with a cocktail consisting of a fluorescently labeled anti-HER2 antibody and an untargeted isotype labeled with a spectrally distinct fluorophore, and imaged using the DDSI imaging protocol. Corresponding clinical histopathology tissue processing (formalin-fixed and paraffin-embedded (FFPE), hematoxylin and eosin (H&E)–stained tissue sections) and anti-HER2 immunohistochemistry (IHC) slides were used to assess the diagnostic performance of the DDSI images. Additionally, diagnostic performance of the DDSI images was compared to the images of the targeted probe alone. Finally, we examined the relationship between DDSI values and HER2 expression levels across specimens.

## Methods and Materials

### Study Procedure

Imaging was performed at the Dartmouth Hitchcock Medical Center (DHMC) in Lebanon, New Hampshire. The clinical study was approved by the Institutional Review Board at Dartmouth College and all procedures followed the approved protocol. Specimen imaging occurred post-operatively in the Department of Pathology and Laboratory Medicine and did not interfere with the standard of care workflow. This study considered mastectomy specimens containing tumor masses > 1 cm in diameter that had not received neoadjuvant chemotherapy. After arrival in the Pathology Department, the fresh specimen was gross-sectioned into tissues approximately 1.5 cm thick, per standard procedure, by a pathologist’s assistant (PA). One section that contained tumor tissue in the cut face was selected for imaging and prepared for DDSI processing. This involved applying a 6-min dual-probe staining protocol described below followed by dual-channel fluorescence imaging. Immediately after acquiring the fluorescence images, a standard red–green–blue (RGB) image under white light illumination was acquired, followed by fixing the breast tissue in preparation for H&E and anti-HER2 IHC staining. Resulting H&E and anti-HER2 IHC stains of each tumor specimen were interpreted by a board-certified breast pathologist (WAW) to report the disease type and HER2 expression level.

### Antibody-Fluorophore Conjugation

The dual-probe staining solution consisted of a fluorescently labeled antibody targeted to HER2 (trastuzumab; Genentech, San Francisco, CA conjugated to IRDye800CW; LI-COR Biosciences, Lincoln, NE) and an untargeted antibody labeled with a spectrally distinct fluorophore (Donkey-anti-Rabbit-IgG conjugated to AlexaFluor 680). The latter was purchased pre-conjugated from Jackson ImmunoResearch Laboratories Inc., (West Grove, PA). The targeted probe was conjugated using the following procedure, which has been reported previously [36].

Lyophilized trastuzumab powder was suspended in 2.5 mL of 8.3 pH phosphate-buffered saline (PBS) to make a 1 mg/mL (6.8 μM) solution of trastuzumab. For fluorophore preparation, IRDye800CW NHS Ester was suspended in anhydrous dimethyl sulfoxide at 10 mg/mL (8.5 mM) stock solution. Then, 9.2 μL of the IRDye800 stock solution was slowly added to the trastuzumab solution and vigorously pipetted to avoid aggregation. The mixture was then shaken at room temperature on a vortex machine at low speed, while protected from light, for 3 h. After this procedure, the antibody-fluorophore mixture was concentrated and buffer-exchanged into 1 × PBS using a 500-μL 10-kDa molecular weight cutoff spin filter spun at 8.5 k revolutions per minute (RPM) for 7 min for multiple cycles. After the buffer exchange was complete, the remaining conjugate was collected by inverting the spin filters into a collection tube and spinning at 1 k RPM for 1 min.

Once the final conjugate was collected, absorbance spectroscopy (Cary 50 Scan UV/Vis Spectrophotometer, Varian, Netherlands) was used to quantify the antibody to fluorophore conjugation ratio. The final conjugate was found to have a dye-to-protein ratio of 2.74 in 2 mL of trastuzumab-IRDye800 conjugation solution. Similarly, absorbance spectroscopy was used to measure the antibody-to-fluorophore conjugation ratio in the pre-made Dk-anti-Rb-IgG-AF680 solution, indicating a dye-to-protein ratio of 0.85.

Staining conjugates were separately aliquoted into vials and stored at − 80 °C until the day of an imaging study. On the day of specimen imaging, the dual-probe staining solution was mixed into a 3-mL solution containing 1 × PBS pH 7, 200 nM antibody concentration of both trastuzumab-IRDye800 and Dk-anti-Rb-IgG-AF680, 1% BSA, and 0.1% Tween 20. Throughout the clinical study period, the concentration of each fluorophore-antibody conjugate was routinely checked on the absorbance spectrophotometer to ensure stable dye-to-protein conjugate binding.

### Dual-Probe Specimen Imaging Procedure and Processing

**Fig. 1:**
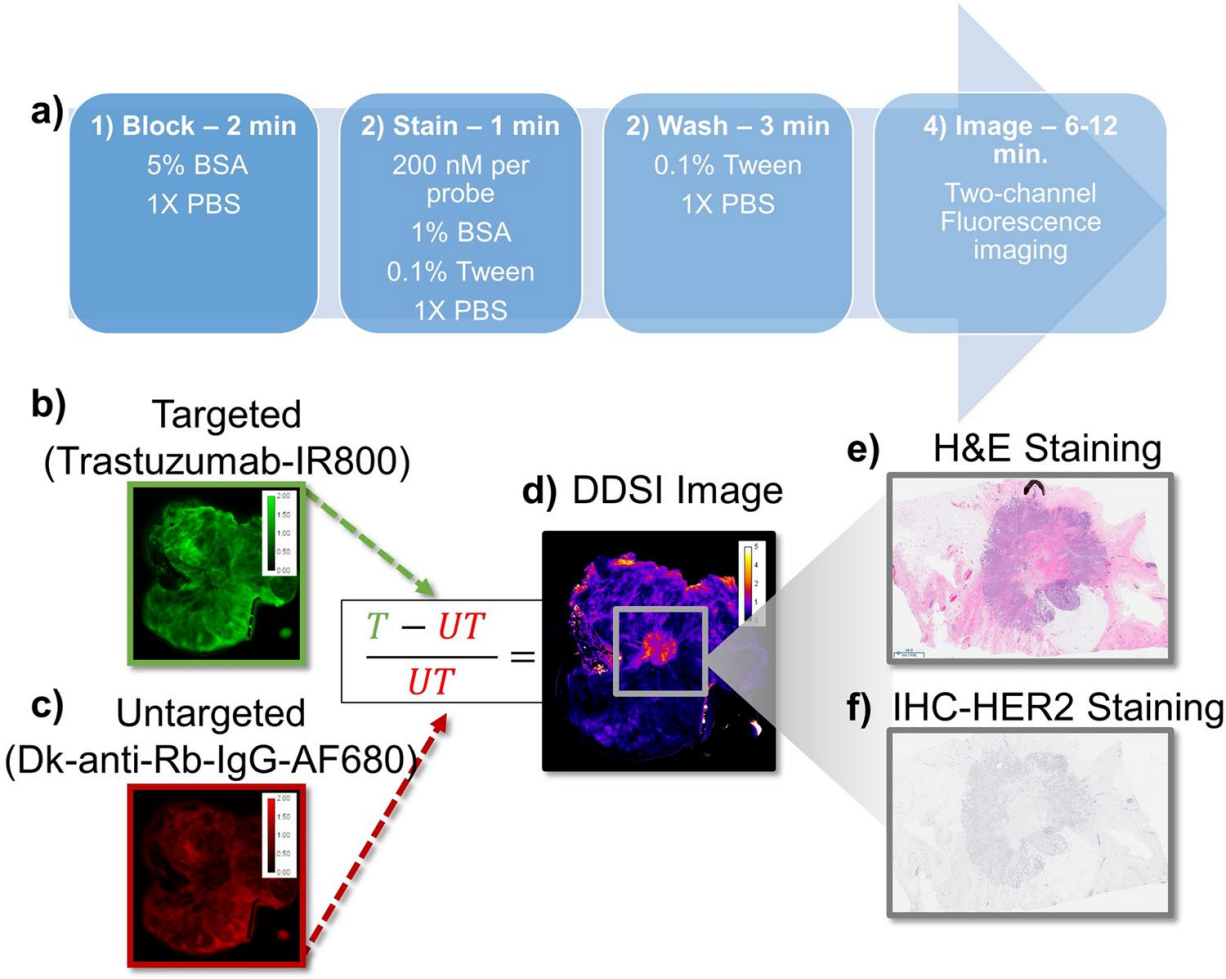
**a** Flowchart of the dual-probe staining and imaging procedure. Examples of (**b**) targeted and (**c**) untargeted fluorescence images which are mathematically compared to create (**d**) the DDSI image. Sections from the imaged surface were also collected and processed for (**e**) H&E and (**f**) anti-HER2 IHC staining

The specimen staining procedure optimized in preclinical models has been previously reported [37] and is outlined here in Fig. 1. Briefly, this procedure consists of submerging a freshly excised specimen in a blocking solution (5% BSA, 1 × PBS) for 2 min, then incubating the specimen for 1 min in the dual-probe staining solution and finally submerging the specimen in a washing solution (1 × PBS with 0.1% Tween 20) for 3 min. Immediately following this procedure, specimens were imaged along with a calibration phantom on an Odyssey CLx Infrared Imaging System (LI-COR Biosciences, Lincoln, NE) in the 700 nm and 800 nm channels at 169-μm resolution. Color images were also acquired on a digital camera for comparison.

The DDSI image processing procedure has been described in multiple previous reports [35, 36]. Briefly, after background correction and confirmation that image intensity met a minimum threshold, each image was normalized to an image of a calibration phantom consisting of the dual-stain cocktail used for that specimen. The DDSI values were then calculated on a pixel-by-pixel basis using the difference between the intensity-normalized targeted and untargeted images divided by the untargeted image (as shown in Fig. 1(d)).

### H&E and Anti-HER2 IHC Processing and Image Analysis

Immediately following DDSI imaging, the imaged specimen was optimally formalin-fixed and paraffin-embedded for sectioning and staining, per standard clinical protocols. H&E and anti-HER2 IHC staining was performed by DHMC Pathology Shared Resources. Serial sections of the tissue face imaged using DDSI were collected for staining after facing the blocks, per standard protocol. Immunohistochemical staining for HER2 was performed using PATHWAY anti-HER-2/*neu* (4B5) antibody (Ventana Medical Systems, Oro Valley, AZ). H&E and anti-HER2 IHC slides were imaged using the Aperio ScanScope slide scanner (Leica Biosystems, USA). Color images were obtained at 0.5 × magnification and exported for further analysis as uint8 RGB TIFF files using the Aperio ImageScope viewing software.

The lead pathologist (WAW) scored each anti-HER2 IHC slide according to the 2018 ASCO/CAP guidelines [41] which scores tumor staining into broad categories of 0, 1 + , 2 + , and 3 + . To further quantify HER2-receptor expression levels, we processed the anti-HER2 IHC pathology images using a previously published protocol [42]. Briefly, for each mastectomy specimen, five sampled ROIs of 600 × 600 μm were selected from tumoral regions of the anti-HER2 IHC image. Each selected region underwent the following procedures using ImageJ and following the step-by-step procedures previously described [42]:

1. Perform color deconvolution using ImageJ’s Color Deconvolution function with the H DAB vector option selected.
2. Threshold the DAB staining (brown) image to separate the brown signal. The threshold value was kept consistent throughout the entire study.
3. Calculate the percent area of brown signal by taking the average of the binarized image.
4. Threshold the hematoxylin staining (blue/purple) image to separate the nucleus signal.
5. Perform a watershed and dot-counting algorithm to count the number of nuclei present within the image (excluding the image edge regions).
6. Divide the percent area of brown signal (calculated in step 3) by the number of nuclei (calculated in step 5) to generate a HER2 score.
7. Normalize all HER2 scores to the HER2( +) sample drop present within the same anti-HER2 IHC pathology stain. Report resulting values as HER2-level percentages.
8. Report on average and standard deviation in normalized HER2-level percentages among 5 sampled ROIs.

Normalized HER2-levels ranged from 0 to 100%, with 100% representing the anti-HER2 IHC expression seen in the HER2( +) sample drop.

### Image Analysis and Statistics

The image acquisition and pathology process produce the following for each specimen: (1) fluorescence images of the targeted and untargeted probes, the DDSI image, and an RGB image, and (2) H&E and anti-HER2 IHC of the same surface from the fixed tissue. Tissue regions were classified by type— adipose, fibroglandular, tumor, fibrosis, or fibrocystic change (FCC)—by the lead pathologist (WAW) using the RGB images and corresponding H&E pathology slides.

In addition to basic assessment of probe uptake using mean values in tissue-specific regions of interest, receiver operator characteristic (ROC) analysis between tissue types was used as a primary evaluative metric. This analysis approach has been used extensively in our previous preclinical publications [35–39]. Here, MATLAB’s *perfcurve.m* function was used to compute the ROC curves and AUC values for tumor-to-all other tissue types identified in each specimen. Mean AUC values for different imaging approaches were compared statistically using a 2-sample paired *t*-test (α = 0.05) and 95% confidence intervals (CIs) were reported.

## Results

Eleven mastectomy specimens from 10 patients were included in this analysis (one patient received a bilateral mastectomy). Table 1 summarizes the tumor features observed within the 11-specimen cohort. Intermediate to high-grade invasive ductal carcinoma (IDC) was observed in 10/11 specimens while 5 of these 10 cases showed intermingled, non-invasive ductal carcinoma in-situ (DCIS). In one case, extensive DCIS with foci of micro-invasion was present, but no visible IDC mass was identified. Analysis of the pre-operative diagnostic biopsy determined that the majority of specimens (81%) were a luminal A molecular subtype (estrogen receptor ER + and/or progesterone PR + , and HER2 −). Post-operative anti-HER2 IHC analysis showed 10/11 specimens had low HER2 expression levels with incomplete/weak membrane staining in > 10% of tumor cells (corresponding to a pathological score of 1 + , which is clinically considered HER2-negative) and one specimen to have amplified HER2 expression levels, characterized by circumferential membrane staining that is complete/strong in > 10% of tumor cells (score of 3 + , clinically HER2-positive).

**Table 1:** Tumor features observed in mastectomy specimen cohort (*n* = 11)

Disease type (determined by H&E):	Intermediate to high-grade IDC only	High grade IDC + focal DCIS	Intermediate grade IDC + extensive DCIS	Extensive DCIS + micro-invasion
	5 (45%)	4 (36%)	1 (9%)	1 (9%)
Receptor status (determined by biopsy):	Luminal A (HER2 −)	HER2 − enriched (ER − , PR − , HER2 +)	Basal-like (ER − , PR − , HER2 −)	
	9 (81%)	1 (9%)	1 (9%)	
HER2 expression (determined by IHC-HER2):	Amplified HER2 expression (score 3 +)	Low HER2 expression (score 1 +)	No HER2 expression (score 0 +)	
	1 (9%)	10 (91%)	0 (0%)	

**Fig. 2:**
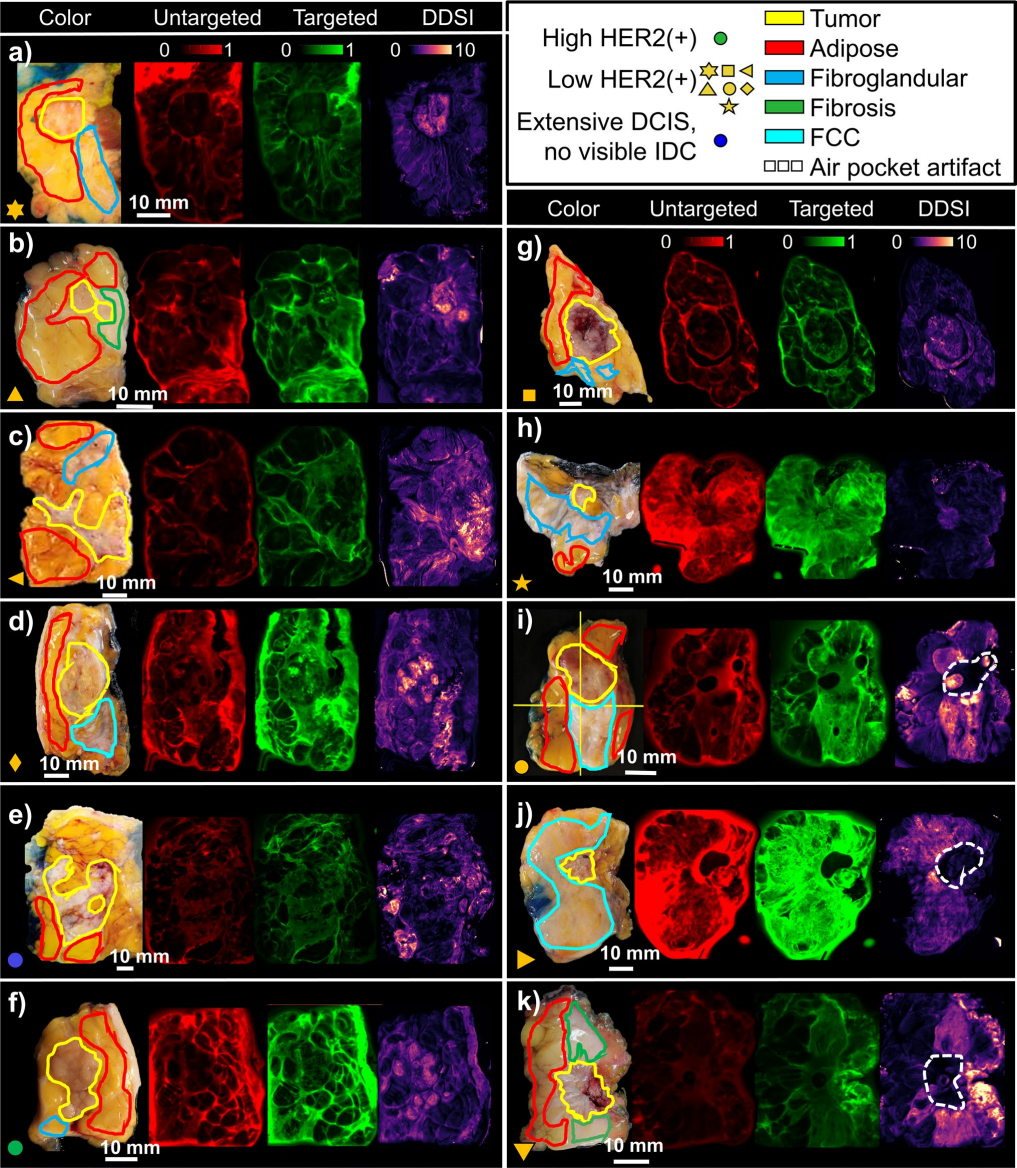
A collection of all fresh specimen images acquired for the study (panels **a**–**k**). Each panel shows the image collection for an individual specimen, and includes the RGB image, targeted probe fluorescence image, untargeted probe fluorescence image, and the DDSI image. Contours overlaid on the RGB image delineate different tissue types, as determined by the lead pathologist. Note that each panel is marked with a symbol that is used throughout the analysis to allow readers to follow individual specimens

**Fig. 3:**
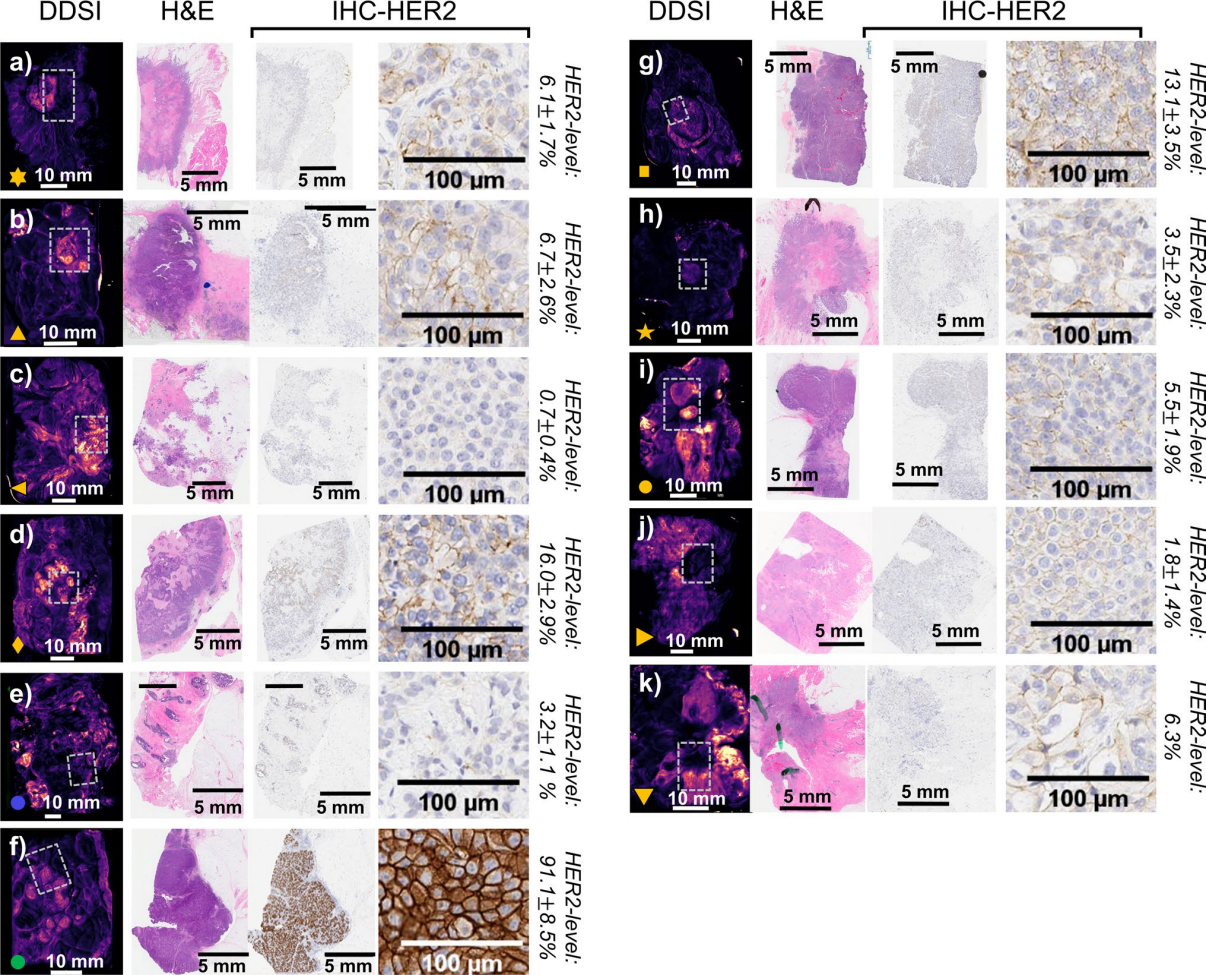
Images of selected pathology slides for each specimen. As in Fig. 2, each panel (**a**–**k**) represents the images for one specimen. The white-dotted rectangles on the DDSI images outline the area of the H&E and IHC slides

Figure 2 shows color images, images of the targeted and untargeted probe channels, and the DDSI image for each specimen. Note that each specimen is labeled with a symbol which is used consistently throughout the results section to enable readers to track individual specimens through the quantitative analysis. Color-coded contours delineating tumor, adipose, fibroglandular, fibrosis, and FCC tissue types are overlaid on the RGB images. Figure 3 provides the corresponding H&E and anti-HER2 IHC slides from sampled tumoral regions in each specimen. The white dotted rectangles overlaid on the DDSI images outline the area of the H&E and IHC slides. Figure 3 also includes the values for the HER2 expression level metric (normalized percent HER2-stained area) computed from the corresponding IHC slides. In specimens *i*, *j*, and *k*, a marked signal drop was observed in a few small regions, caused by an air pocket/bubble between the specimen surface and glass plate of the imaging system. In specimen *i*, the air pocket region was excluded from the full quantitative analysis of the specimen; however, because the artifact coincided with the tumor region in specimens *j* and *k*, these specimens were not included in analyses that required tumor values (ROC analysis).

Qualitative inspection of the images in Fig. 2 reveals several notable observations. First, when considered alone, the targeted probe images show no preferential highlighting of tumor tissue in any of the specimens, and in some cases even appeared to produce lower signal than in surrounding normal tissue. Yet, despite this targeted probe uptake pattern, the DDSI calculation produced visible tumor-to-normal contrast in some of these specimens. This is most obvious in specimens *a*, *b*, *g*, and *h*, which show close qualitative alignment of the DDSI intensity distributions and the tumor region outlines on the corresponding RGB images. Additionally, while DDSI values in the adipose and fibroglandular tissue were relatively low, the DDSI values appear elevated in tissues classified as FCC. Finally, these images reveal that many of the tumors which present with low HER2 expression produced significant DDSI tumor-to-normal contrast.

**Fig. 4:**
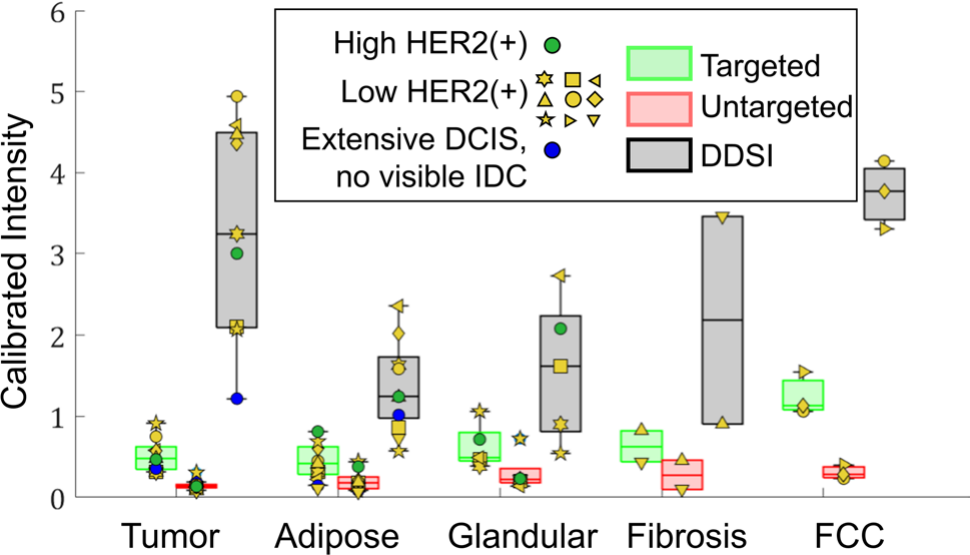
Box and whisker plot of the mean values of the targeted, untargeted, and DDSI images in each tissue region (shown in Fig. 2). Note that the markers reference specific specimens as shown in Fig. 2

Quantitative analysis of these tissue types confirms the qualitative observations. Figure 4 shows the average values in each tissue-type region for the untargeted probe, targeted probe, and DDSI images. Targeted probe uptake was largely consistent across tissue types and showed no enhancement in the tumor regions compared to normal tissue types. Tissues classified as FCC were the exception, and showed enhanced targeted probe uptake compared to other tissue types, including tumor. Unlike the targeted probe images, the average DDSI values were significantly higher in tumor tissue than in adipose and glandular tissue, with *p*-values = 0.002 and 0.013, respectively. The DDSI values in FCC tissues remained high with mean values similar to tumor (*p* = 0.79). Further examination of Fig. 4 reveals that the specimen containing extensive DCIS with foci of micro-invasion and no IDC mass produced the lowest tumor-region DDSI values. Because the tumor was ill-defined in this specimen, the contoured tumor region considered for analysis encompassed a heterogeneous mixture of cell phenotypes, possibly explaining the low DDSI contrast. The specimen containing the tumor with the highest HER2 expression level, the only score 3 + tumor in the study, did not produce the highest tumor DDSI values. Indeed, DDSI values were higher in a majority of the tumors with low HER2 expression levels included in the analysis.

**Fig. 5:**
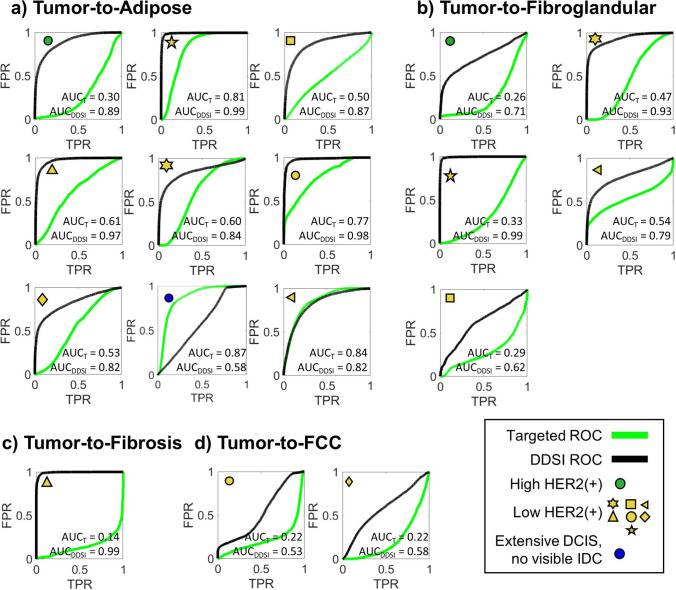
Receiver-operator-characteristic curves from the targeted and DDSI images computed for tumor to several other tissue types. All specimens that had a defined IDC and no interfering air pocket artifacts are included

To examine the capacity of the staining techniques to distinguish tumor tissue, ROC analysis comparing tumor to other tissue types was completed for each specimen (excluding specimens *j* and *k* per the reasons cited above). This was accomplished by computing the ROC metrics on all pixels in the contoured regions shown in Fig. 2. Figure 5 shows the ROC results for the targeted probe and DDSI images for each specimen, along with the corresponding AUC values. All specimens considered contained adipose tissue, while only subsets of specimens contained glandular (*N* = 5), FCC (*N* = 2), and fibrosis (*N* = 1).

Examination of these graphs indicates that the DDSI method increased the tumor-to-adipose AUC compared to targeted probe images alone in seven of the nine specimens considered. In eight of the nine specimens, the DDSI tumor-to-adipose AUC values were equal to or greater than 0.82, and as high as 0.99. The outlier specimen, which produced an AUC = 0.58, was the sole specimen diagnosed as extensive DCIS with foci of micro-invasion (Fig. 2(e)), which again could be explained by the heterogeneity in the contoured tumor region described above. The largest increase in AUC was observed for the only specimen scored a 3 + for HER2 expression (Fig. 2(f)). Of the two specimens that showed lower DDSI AUC compared to the targeted probe images, one produced very similar AUC performance (AUC = 0.84 and 0.82 for DDSI and targeted images, respectively), and the other was in the extensive DCIS specimen. Tumor-to-glandular AUC values also showed substantial increases between the targeted and DDSI images, though the resulting DDSI values were more variable (ranging from 0.62 to 0.99). Notably, the targeted image AUC values were all below 0.5 before application of the DDSI processing step. Finally, DDSI processing increased the tumor-to-FCC and tumor-to-fibrosis AUC values compared to targeted imaging alone in the few specimens that presented with these tissues. The latter showed the largest change, with the targeted probe and DDSI images producing AUC values of 0.13 and 0.99, respectively.

**Fig. 6:**
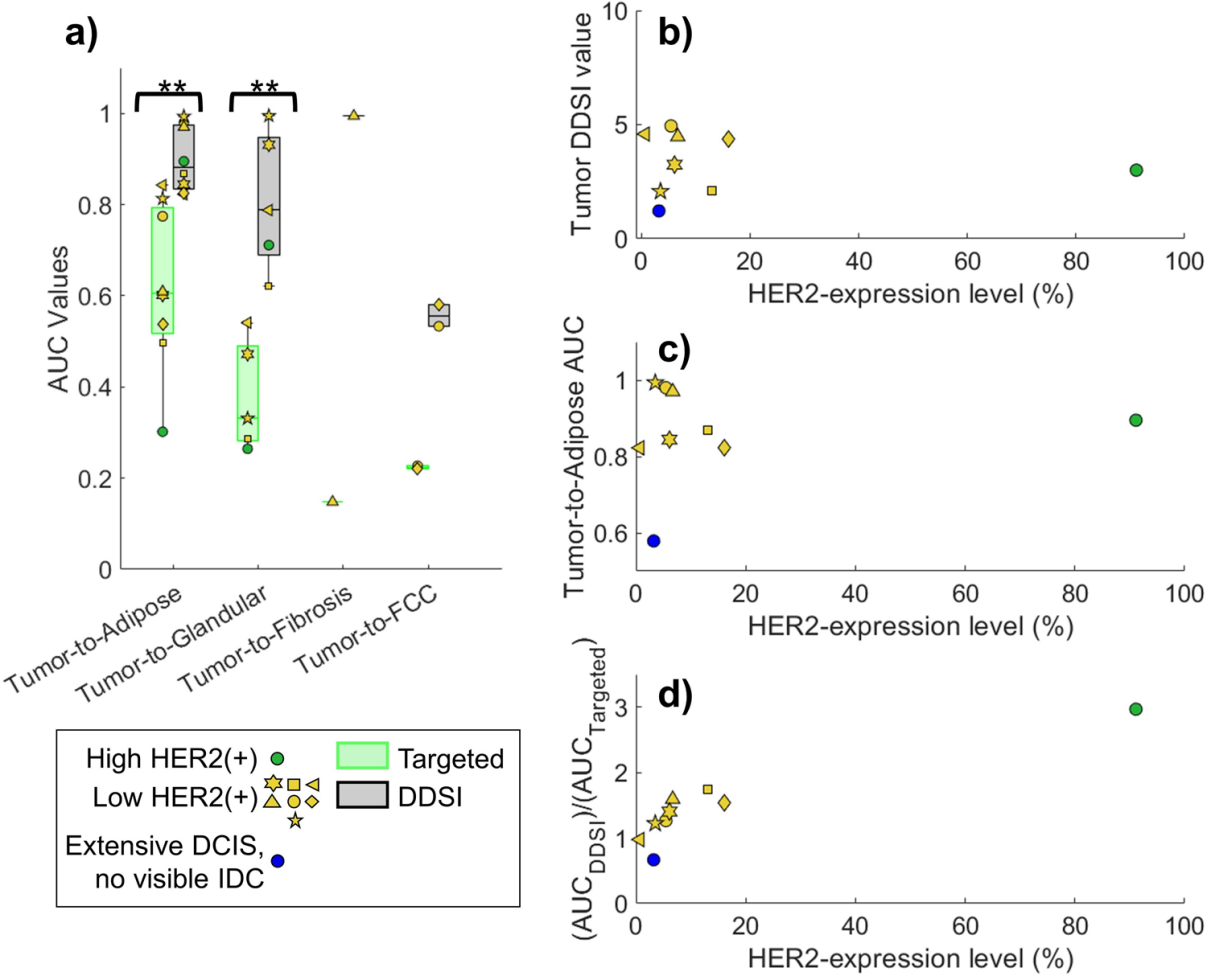
**a** Box and whisker plots of ROC-AUC values for targeted and DDSI images for all specimens that had a defined IDC and no interfering air pocket artifact. **b** DDSI values in tumor plotted as a function of HER2 expression level computed from the IHC images. **c** Tumor-to-adipose DDSI AUC values plotted as a function of HER2 expression level. **d** The ratio of (AUC_DDSI_)/(AUC_Targeted_) for tumor-to-adipose tissue plotted as a function of HER2 expression

Figure 6(a) shows a box and whisker plot of the AUC values for the specimens containing identifiable IDC masses presented in Fig. 5 (this excludes the DCIS specimen that presented with no identifiable IDC mass). The mean tumor-to-adipose AUC values were 0.62 ± 0.18 and 0.90 ± 0.07 for the targeted probe and DDSI images, respectively, and the mean tumor-to-glandular AUC values were 0.38 ± 0.12 and 0.81 ± 0.15 for targeted probe and DDSI images. The increase in AUC performance provided by DDSI over the targeted probe imaging was statistically significant in both cases, with *p* = 0.003 (95% CI = [− 0.43, − 0.13]) for tumor-to-adipose AUC and *p* = 0.004 (95% CI = [− 0.62, − 0.23]) for tumor-to-fibroglandular AUC. Although DDSI also appeared to improve tumor-to-FCC and tumor-to-fibrosis AUC performance, statistical comparisons were not performed due to the limited sample number. A notable observation is that even with DDSI imaging, the tumor-to-FCC AUC value was relatively low (AUC = 0.56).

To investigate whether the DDSI values capture information about expression level, the recovered HER2 expression levels, determined by anti-HER2 IHC, were compared to the recovered DDSI values. Figure 6(b) plots HER2 expression level vs. DDSI value in the tumor, showing no obvious correlation between the two. A similar, non-correlative result was obtained when plotting HER2 expression level vs. DDSI tumor-to-adipose AUC values (Fig. 6(c)). Finally, we computed the (AUC_DDSI_)/(AUC_Targeted_) ratio and plotted this factor against HER2 expression level (Fig. 6(d)). This analysis suggests that the factor increase in AUC values realized by applying the DDSI method is related to the expression level of the tumor.

## Discussion

The multi-probe normalization imaging approach, here termed DDSI, was developed to identify tumor cells in fresh specimens using a rapid receptor-specific fluorescence staining and imaging protocol. Prior efforts in our lab have demonstrated high tumor sensitivity and specificity in several preclinical models expressing different tumor-associated receptors [35–39], and have been used to optimize the protocol for efficiency [37]. Researchers from Dr. Jonathan Liu’s group have also made important contributions to the field, examining similar tissue staining and image processing strategies using SERS particle labels instead of fluorophore labels [26–28]. As part of that effort, Wang et al. reported normalized receptor imaging of multiple biomarkers in clinical breast specimens [28]. To our knowledge, the study presented herein is the first to report DDSI on fresh clinical specimens using fluorophore-based probes.

In this preliminary clinical study, the DDSI approach showed encouraging diagnostic performance. We observed mean tumor-to-adipose AUC values of 0.90 ± 0.07 in all specimens with IDC masses (excluding two specimens with air pocket artifacts), including all specimens with low HER2 expression (score of 1 +). These values are generally consistent with those reported in our preclinical studies [35–39]. Because fibroglandular tissue volumes in mouse models are small and difficult to identify, we have not previously reported on tumor-to-fibroglandular DDSI performance. In the clinical specimens examined herein, we observed mean DDSI tumor-to-fibroglandular AUC values of 0.81 ± 0.15, which suggests that distinguishing tumor from glandular tissue will be more challenging than from adipose tissue. Further evaluating DDSI behavior in glandular tissue will be an important consideration in future studies.

The relationship between DDSI values receptor expression levels reported herein is intriguing. We observed no correlation between tumor DDSI values and HER2 receptor expression, or between AUC performance and receptor expression. Relatedly, the diagnostic performance was robust even in low expressing tumors traditionally classified as clinically HER2-negative (score 1 +). Although initially surprising, our previous preclinical studies suggested that DDSI values may not be linearly related to receptor expression [38], and that once an expression level threshold is reached, AUC does not change much with higher expression levels. These results come in the context of dramatic reports showing that new HER2-targeted antibody–drug conjugates are highly effective in a large percentage of breast tumors traditionally classified as HER2-negative (score 1 +) [43, 44]. Taken together, these results suggest that HER2-targeted imaging for tumor identification may be applicable in a much wider patient population than anticipated.

Although only present in a limited number of specimens, tissues classified as FCC produced elevated DDSI values, reducing tumor-to-FCC contrast of the DDSI signal. In the current study, the elevated values were largely driven by preferential uptake of the targeted stain in these tissues. This could present a challenge for clinical translation of the technique, potentially requiring the deployment of methods to either identify FCC or otherwise increase tumor specificity, including adding other stains that label FCC or other tumor markers, or investigating endogenous optical techniques, such as scatter-based imaging [15, 16] that may be able to distinguish FCC.

Another central finding is that applying the DDSI method significantly increased tumor-to-normal tissue contrast and diagnostic performance compared to staining with a targeted imaging probe alone, again consistent with the bulk of our previous preclinical work. The extent to which the DDSI method improved diagnostic performance was quite striking, improving mean AUC values by 0.28 and 0.43, depending on tissue type comparisons. Interestingly, although we observed no correlation in DDSI values vs. expression level, the improvement in AUC values between DDSI and targeted-probe-alone imaging appears related to expression levels. This suggests that the DDSI approach is more adept at emphasizing receptor expression differences than staining with a single targeted probe.

Although the results presented here are largely encouraging, it should be recognized that this preliminary clinical study was performed on cut surfaces of mastectomy specimens with grossly identifiable tumor regions. Identifying small residual tumors on BCS specimen surfaces will certainly be more challenging, and will require rigorous image/tissue registration for validation. Understanding the sensitivity and specificity limits of the approach in BCS margins will be a central focus of future evaluation. Nonetheless, this study helps establish the feasibility of deploying the approach for clinical specimen imaging, and provides valuable guidance for continued development.

## Conclusion

Accurate and rapid assessment of margin status in surgical specimens during surgery would help facilitate complete tumor removal, potentially reducing repeat surgery rates. Herein, we present the first experience with our fluorophore-based dual-probe topical staining approach in clinical breast specimens. Consistent with preclinical results, high diagnostic performance between tumor and the most common normal tissues was observed, though this performance dropped in the presence of fibrocystic changes. Notably, specimens containing tumors that would traditionally be classified as HER2-negative showed high ROC performance as a consequence of their low, but measurable, HER2 expression, suggesting that HER2-based imaging could be effective in patients beyond those with tumors defined as HER2-positive based on current clinical standards. These encouraging results support further development and evaluation of this promising approach.
